# Molecular Architecture of the H2AX-Containing Nucleosome from Arabidopsis

**DOI:** 10.3390/cells15191795

**Published:** 2026-10-01

**Authors:** Rashmi Panigrahi, Ross Edwards, Nathaniel Agbagba, Mark Glover

**Affiliations:** 1 Department of Human Biosciences, Memorial University of Newfoundland, St John’s, NL A1C 5S7, Canada; 2 Department of Medical Microbiology and Immunology, University of Alberta, Edmonton, AB T6G 2E1, Canada; 3 Department of Biochemistry, University of Alberta, Edmonton, AB T6G 2H7, Canada

**Keywords:** *Arabidopsis thaliana*, nucleosome, DNA damage, S phase, cryogenic electron microscopy

## Abstract

**Highlights:**

**Main findings**

- We report the first cryo-EM structures of Arabidopsis thaliana nucleosomes containing replication-associated histones together with the DNA damage-responsive H2AXa variant.
- The distinct plant histone composition preserves the canonical nucleosome architecture and supports conserved but structurally distinct modes of nucleosome packing.

**Implications**

iii. Our work indicate that histone sequence divergence in plants can be accommodated without disrupting the nucleosome core.
iv. Our work provides a structural basis for studying H2Xa in plant DNA damage response.

**Abstract:**

Nucleosomes integrate diverse histone variants to organize chromatin and coordinate genome maintenance. In plants, the structural interplay between replication-associated histones and DNA damage-responsive histone variants remains poorly understood. Here, we determine cryogenic electron microscopy structures of an *Arabidopsis thaliana* (*At*) nucleosome containing the replication-associated histones H2B.6 and H3.1 together with the DNA damage-responsive variant H2AXa. The mononucleosome structure, resolved at 2.73 Å, preserves the canonical nucleosome architecture. Unexpectedly, the reconstituted mononucleosomes formed defined higher-order assemblies in the absence of linker DNA or chemical crosslinking. Two distinct nucleosome–nucleosome arrangements, parallel and right offset, were identified. The right offset closely resembles the human counterpart, whereas the parallel arrangement exhibits slight displacement of H2B structural elements at the inter-nucleosomal interface. Together these structures reveal how a distinct histone composition preserves the canonical nucleosome architecture while supporting multiple defined modes of nucleosome packing, providing structural insights into conserved and divergent features of H2AX-containing chromatin organization across plants and animals.

## 1. Introduction

Plant genomes are persistently challenged by factors such as ultraviolet and ionizing radiation, as plants cannot move away from unfavorable conditions and must rely on sunlight as their primary energy source. In addition, elevated soil salinity, drought and dehydration, chemical mutagens, and reactive or alkylating molecules produced during normal cellular metabolism represent additional environmental stressors to which the plant is continuously exposed [1,2,3]. These stressors generate diverse forms of DNA lesions, including base damage, DNA crosslinks, and single- and double-strand breaks (DSBs) [4]. Maintaining genome integrity under these conditions is essential for plant growth, development, and adaptation. Protecting the genome depends not only on DNA repair pathways but also on the chromatin environment in which DNA is packaged.

Throughout evolution, the nucleosome has remained a defining hallmark of eukaryotic genomes. It serves as the fundamental structural unit of chromatin and consists of approximately 145 base pairs of double-stranded DNA (dsDNA) wrapped around a histone octamer containing two copies each of the core histones H2A, H2B, H3, and H4. Beyond providing a compact organization of genomic DNA, nucleosomes regulate the accessibility and organization of DNA and contribute to the higher-order chromatin architecture [5,6], primarily driven by inter-nucleosomal interactions [7,8]. While histone H4 is highly conserved in plants, the other three core histones, H2A, H2B, and H3, are encoded by multiple genes that generate histone variants with distinct sequence features and biological functions, with some variants exhibiting cell-cycle-dependent expression patterns [9,10,11]. These histone variants act as non-allelic isoforms of their canonical counterparts and can replace canonical histones at specific chromatin loci, thereby contributing to nucleosome stability and enhancing the structural diversity and functional plasticity of chromatin.

The dynamic organization of chromatin not only regulates gene expression but also plays an essential role in the cellular response to DNA damage. Histone diversity is particularly important during the S phase, when DNA replication creates a heightened risk of replication-associated DNA lesions and requires coordinated regulation of DNA replication, chromatin reorganization, and DNA repair. In plants, chromatin compaction contributes to genome stability by regulating the accessibility of DNA repair factors to DSB lesions while preserving chromosome integrity during repair. Among the various types of DNA lesions, DSBs are particularly lethal because they sever both strands of the DNA helix and physically disrupt chromosome continuity [3,4]. If these breaks are not properly repaired, extensive segments of genetic material can be lost. When such damaged chromosomes are passed on during cell division, the resulting daughter cells inherit incomplete genomes, which can seriously impair their viability and function. In response to DSBs, phosphatidylinositol 3-kinase-related kinases (PIKKs), such as the Ataxia Telangiectasia Mutated (ATM) kinase, Ataxia Telangiectasia and Rad3-related (ATR) kinase, and, in many organisms, DNA-dependent protein kinase catalytic subunit (DNA-PKcs), rapidly phosphorylate the histone variant H2AX at its conserved C-terminal SQ motif [12]. The phosphorylation of serine on the SQ motif initiates DNA damage signaling by creating a platform that recruits DNA repair proteins and activates cell cycle checkpoints, allowing time for DNA repair. The resulting phosphorylated form of H2AX, known as γH2AX, serves as a central and highly sensitive biomarker of DSBs [13]. While the mechanisms of DNA damage sensing by the ATM and ATR kinases are conserved between plants and animals [2], DNA-PKcs homologs have been identified in green plants ranging from microscopic green algae to gymnosperms but have not been detected in angiosperms [14]. The absence of DNA-PKcs in angiosperms suggests that certain aspects of the non-homologous end-joining (NHEJ) pathway, and DNA damage signaling, have diverged during plant evolution. In *Arabidopsis thaliana*, H2AX is represented by two isoforms, H2AXa and H2AXb [15,16]. While H2AXb participates in both homologous recombination (HR) and NHEJ pathways, H2AXa is specifically involved in HR, a DNA repair pathway that is particularly relevant during the S phase.

Despite its central role in DNA damage signaling, the structural basis of H2AX-mediated chromatin regulation remains incompletely understood, particularly in plants. In our previous study, we determined the cryo-electron microscopy (cryo-EM) structures of both human H2AX- and γH2AX-containing nucleosomes and demonstrated that phosphorylation of the H2AX C-terminal tail preserves the overall nucleosome architecture while modulating inter-nucleosomal interactions through the H4 N-terminal tail [13]. These findings established a structural framework for understanding how H2AX phosphorylation contributes to chromatin organization during DNA damage signaling in mammals. However, whether these structural features are conserved in plants has remained unknown.

**Figure 1:**
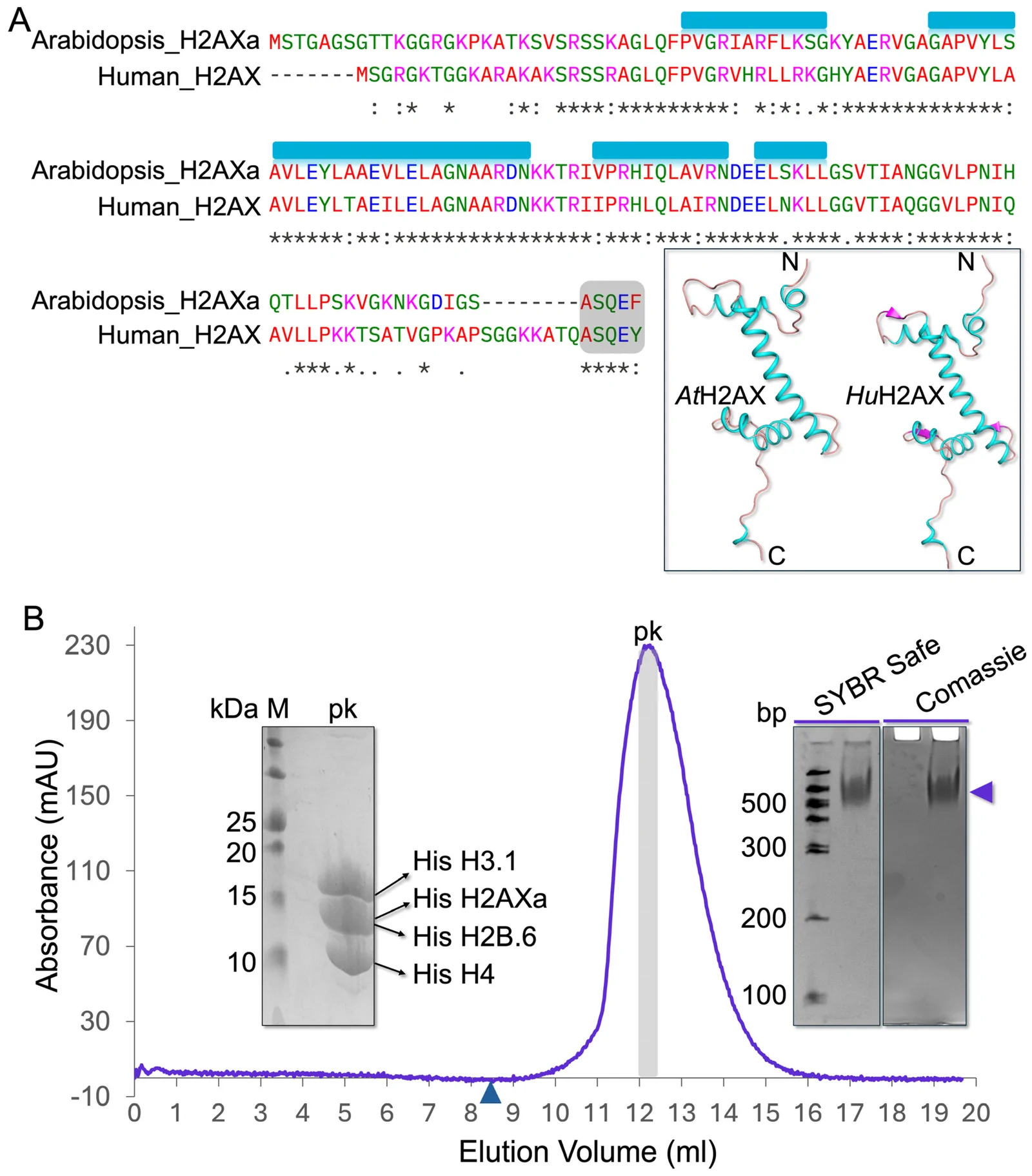
Reconstitution of the *Arabidopsis* H2AXa-containing nucleosome. (**A**) Sequence alignment of *Arabidopsis thaliana* H2AXa and human H2AX. The conserved SQ motif is highlighted, and the structural comparison of the corresponding H2AX proteins is shown on the right. (**B**) The size-exclusion chromatography profile of the co-expressed Arabidopsis histone complex. The inset shows SDS-PAGE analysis confirming the presence of H2AXa, H2B.6, H3.1, and H4 in the purified complex. The reconstituted NCP was analyzed by native PAGE, with the NCP band indicated by the arrowhead.

In this study, we present the cryo-EM structures of the *Arabidopsis thaliana* H2AXa-containing nucleosome core particle (*At*H2AX NCP), assembled with the replication-associated histones H2B.6 and H3.1, providing the first structural insights into a plant H2AX-containing nucleosome assembled with these histone variants. The sequences of H2AX from human and Arabidopsis differ in specific regions (Figure 1A). The nucleosome structures from Arabidopsis enable direct comparison with our previously determined human H2AX-containing nucleosome structures, providing a structural framework to assess conserved and divergent features of H2AX-containing chromatin organization across plants and animals.

## 2. Materials and Methods

### 2.1. Preparation of Octamer Containing H2Xa and Replication-Associated Histones H2B.6 and H3.1 from Arabidopsis

The full-length cDNA of *Arabidopsis thaliana* histones H2AXa (HTA5), H2B.6 (HTB9), H3.1 (HTR1), and H4, with UniProt accession IDs O04848, O23629, P59226, and P59259, respectively, were cloned into Duet vectors and overexpressed in BL21 (DE3) pLysS cells. Histone purification was subsequently performed as described previously [13].

Specifically, the transformant colonies were inoculated into Luria–Bertani broth (Fisher Scientific, Nepean, Canada), and these starter cultures were grown overnight at 37 °C. Large-scale cultures were inoculated with the starter cultures and grown until the OD600 reached ~0.6. Histone expression was induced by adding 0.5 mM isopropyl β-D-1-thiogalactopyranoside (IPTG) followed by incubation for 12 h at 18 °C. Cells were harvested by centrifugation at 4500 rpm (approximately 3880× *g*) for 15 min at 4 °C, using a Beckam Coulter Avanti J-25 centrifuge (Beckman Coulter Life Sciences, Brea, CA, USA) equipped with a JLA-8.1 rotor. Cell pellets were stored at −80 °C until purification.

For purification, cell pellets were resuspended in lysis buffer containing 20 mM Tris-HCl (pH 7.0), 2.0 M sodium chloride, and 1 mM phenylmethanesulfonylfluoride and lysed using an EmulsiFlex-C3 high-pressure homogenizer (Avestin, ON, Canada). The lysate was clarified using centrifugation. The clarified lysate was loaded onto a HisTrap^TM^ Fast Flow column (Cytiva, MA, Canada) connected to an AKTA Purifier chromatography system. Bacterial protein contaminants were removed using increasing concentrations of imidazole in the lysis buffer (0–1 M), with the target protein eluting at 500 mM imidazole. The purified histone octamer was subsequently loaded onto a Superdex 200 10/300 GL column (Cytiva, MA, Canada) pre-equilibrated with degassed lysis buffer. The histone octamer peak was eluted at a retention volume of 12.0 mL. Peak fractions were pooled and concentrated to 20 μM, aliquoted, and flash-frozen in the presence of 50% glycerol for storage.

### 2.2. 601–145 bp DNA Preparation

The 145 bp Widom-601 DNA was prepared on a preparative scale following a previously reported procedure [13,17]. Briefly, the 601–145 bp fragment was excised from the pUC57 plasmid containing six tandem copies of the 601–145 bp sequence by EcoRV digestion, followed by PEG precipitation. The PEG-precipitated DNA was redissolved, followed by a phenol–chloroform extraction. The DNA-containing aqueous layer was pooled and subjected to ethanol precipitation. DNA purity was assessed at each purification step by electrophoresis on a 1.5% agarose gel. Following a final size-exclusion chromatography step, the purified 601–145 bp DNA was concentrated to 1 mg/ml and used for nucleosome assembly.

### 2.3. Nucleosome Reconstitution

*Arabidopsis* histone-containing nucleosomes were assembled following a previously established protocol with minor modifications [13]. The purified histone octamer was combined with DNA at an octamer-to-DNA molar ratio of 1.2:1.0. The mixture was subjected to stepwise salt dialysis using 10 mM Tris-HCl (pH 7.0) and 0.5 mM TCEP as the base buffer, with NaCl concentrations subsequently reduced from 1 M to 800 mM, 500 mM and finally 150 mM NaCl. Each dialysis step was performed for 5–6 h. Successful assembly of *Arabidopsis* histone-containing nucleosomes was assessed by electrophoresis on 5% native polyacrylamide gels. The DNA-containing species were visualized by staining the gel with SYBR Safe DNA gel stain (Thermo Fisher Scientific, Mississauga, ON, Canada).

### 2.4. Cryo-EM Grid Preparation

For grid preparation, 3 μL of *Arabidopsis* histone-containing nucleosomes at a concentration of 3 μM was applied to 300-mesh Quantifoil R2/2 copper grids following 60 s of glow discharge using a Solarus plasma cleaner (Gatan, Pleasanton, CA, USA). The grids were vitrified by plunge-freezing in liquid ethane using an FEI Vitribot (Thermo Fisher Scientific, Waltham, MA, USA) at 4 °C and 100% humidity, with a blot time of 0–5 s and a blot force of 5.

### 2.5. Cryo-EM Data Collection and Processing

Cryo-EM images were acquired on a Thermo Fisher Scientific Titan Krios G2 microscope operated at 300 KeV, featuring a Selectris X energy filter and a Falcon 4i Ceta-D electron detector. Movies were acquired in energy-filtered mode with an energy slit of 20 eV. The zero-tilted collection was collected with EPU (Thermo Fisher Scientific, Waltham, MA, USA), whereas the 30-degree tilted data were collected using SerialEM. Cryo-EM data processing was carried out in cryoSPARC v4.2.1 [18]. Details of data collection are provided in Table 1. For model building, the cryo-EM structure of the canonical human H2AX-containg nucleosome (PDB: 9MMK) was used. The individual residues were altered to correspond to the sequences of *Arabidopsis* H2AXa, H2B.6, H3.1, and H4, and rigid-body fitting was performed using ChimeraX [19]. We built the nucleosome model into a map using iterative rounds of model building and local refinement in Coot [20] and global real-space refinement in Phenix [21], leading to the final models. The canonical *At*H2Axa-containing nucleosome structure was used to build the stacked nucleosome model. The N-terminus of H4 linker conformation adjustment was aided by ISOLDE [22]. Graphics were generated in ChimeraX and PyMol [23]. Local resolution was plotted with Resmap [24].

## 3. Results

### 3.1. Reconstitution of AtH2AXa-Containing NCPs with Replication-Associated Histones

The S phase is characterized by active DNA replication and represents a period of increased vulnerability to replication-associated DNA damage [25,26,27]. H2B.6 and H3.1 are predominantly synthesized and deposited during replication [28,29], whereas H2AXa is a DNA damage-responsive variant involved in the cellular response to DNA double-strand breaks [15]. Given the distinct roles of these histones during DNA replication and the DNA damage response, we sought to determine how their incorporation into the same nucleosome particle influences its structure and inter-nucleosomal organization. To investigate this question, we employed a co-expression strategy based on the Duet vector system to simultaneously express the four *Arabidopsis* core histones in *E. coli*. The resulting histone octamer comprised two copies each of histone variants H2AXa, H2B.6, H3.1, and H4, forming an H2Xa-containing *At*NCP assembled with replication-associated H2B.6 and H3.1.

The co-expressed histones were purified using Ni-NTA affinity chromatography, followed by size exclusion to obtain monodispersed complexes, and analyzed by SDS-PAGE, which confirmed the presence of all four histones at their expected molecular masses (Figure 1B). The purified histone complex was subsequently used for nucleosome reconstitution with the defined Widom 601 (601–145 bp) nucleosomal DNA [5]. Following reconstitution, the resulting *At*NCPs were analyzed by native polyacrylamide gel electrophoresis, and well-resolved NCP fractions without free/unwrapped DNA were used for subsequent cryo-EM studies (Figure 1B inset).

### 3.2. Structural Analysis of Mononucleosomes from Arabidopsis

The *Arabidopsis* core histones exhibit varying degrees of sequence conservation relative to their human counterparts. *Arabidopsis* H3.1 and H4 are highly conserved, with *Arabidopsis* H3.1 differing from the human sequence at only four residues (Y42F, R53K, M91A, and C97A), while H4 differs at two residues (V61I and K78R). In contrast, greater sequence divergence is observed for H2B.6 and H2AXa. Replication-coupled *Arabidopsis* H2B.6 [30] shares 68% sequence identity with human H2B type 1-J [31] and contains an additional 34 residues at its N-terminus, resulting in a extended N-terminal tail. H2AXa also exhibits sequence divergence within both its N- and C-terminal regions relative to human H2AX, although the SQ motif, characteristic of H2AX, is conserved in both *Arabidopsis* and humans (Figure 1B). Despite these sequence differences, the high conservation of histone-fold domains suggests that the fundamental architecture required for histone–histone interactions and DNA association is preserved.

**Figure 2:**
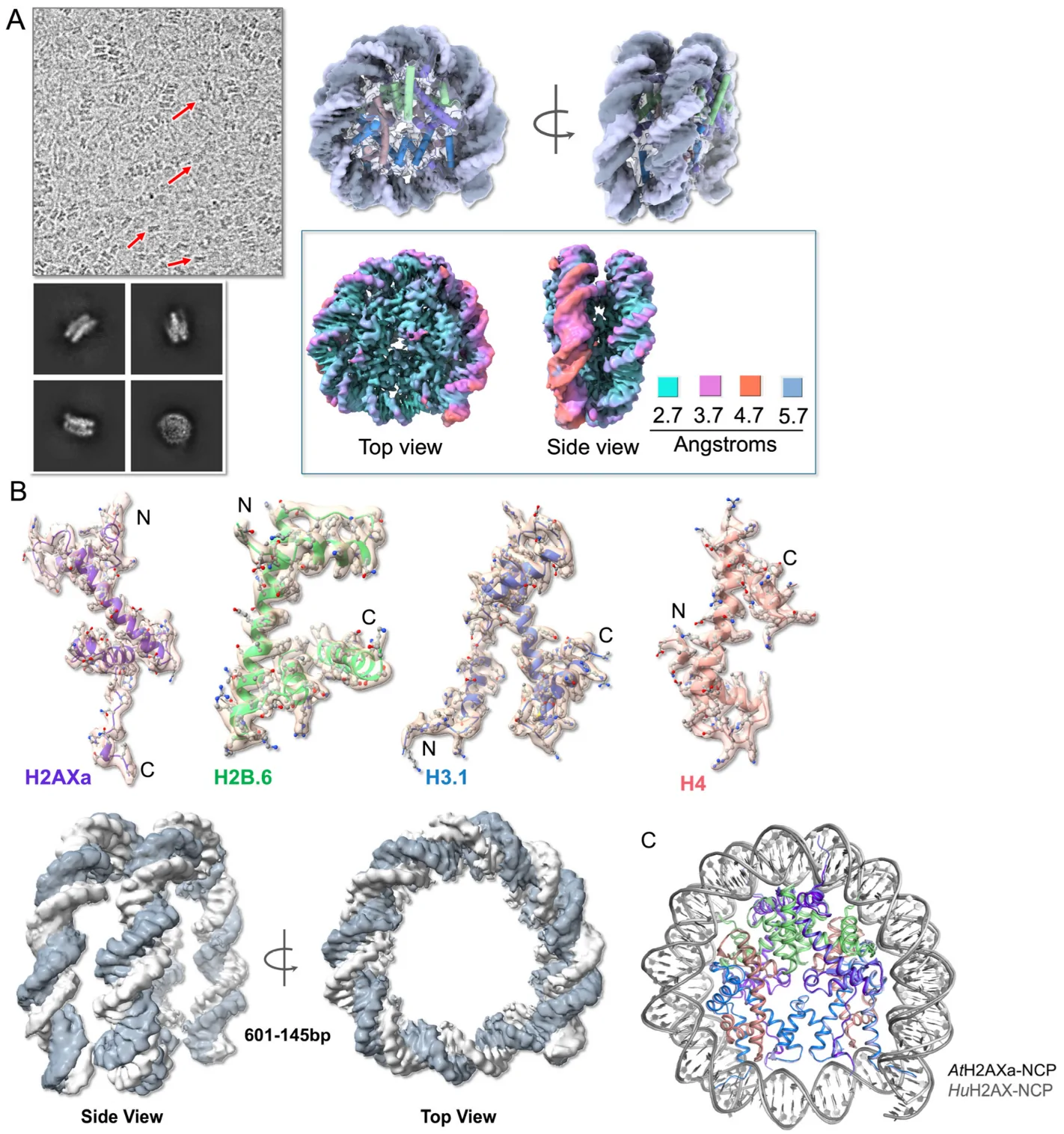
Cryo-EM structure of *At*NCP canonical structure. (**A**) Representative micrograph and 2D class averages of the reconstituted mononucleosome. Cryo-EM density with the refined model is shown in the top and side views, with the local resolution scale indicated. (**B**) The atomic model of the *At*NCP fitted into the cryo-EM density, showing H2AXa, H2B.6, H3.1, and H4. The corresponding histone models are shown with their resolved residues. Cryo-EM density of the 601–145 bp DNA in side and disk views are shown. (**C**) Structural comparison of the *At*NCP with the previously determined human NCP shown as an overlay.

To determine whether these sequence differences alter the overall nucleosome architecture, we collected cryo-EM datasets of the reconstituted *At*NCP at 0° and 30° tilts. Iterative 2D and 3D classifications enabled particle averaging across multiple orientations, including disk-like, tilted, and side views. The *At*NCP mononucleosome’s canonical structure was reconstructed at a nominal resolution of 2.73 Å, as determined by the gold-standard FSC 0.143 criterion, and closely resembles the human H2AX-containing NCP (PDB: 9MMK) (Figure 2A, Supplementary Figure S1A). The 601–145 bp dsDNA wraps around the histone octamer in a left-handed superhelical trajectory. The cryo-EM density supported model building for residues Ser22-Pro125 of H2AXa, Ser59–Ser150 of H2B.6, Lys38-Ala136 of H3.1, and Asp25-Gly103 of H4 (Figure 2B). The histone tails, including the C-terminal region containing the SQ motif of H2AXa, were not resolved, consistent with their intrinsic conformational flexibility. The histone core maintains extensive contacts with the negatively charged DNA along the nucleosomal DNA gyres. Multiple residues from H2Xa, H2B.6, H3.1, and H4 contribute to the histone–DNA interaction, including Ser22, Val 23, Arg25, Lys28, Arg40, Arg50, Val51, Ala53, Lys83, and Arg85 from H2AXa; Ser59, Val60, Tyr66, Ser82, Lys112, and Thr114 from H2B.6; Lys38, Arg41, Phe42, Arg43, Gly45, Thr46, Val47, Ala48, Arg64, Lys65, Leu66, Arg73, Phe85, Gln86, Val118, and Thr119 from H3.1; and Arg36, Arg 37, Ile47, Ser48, Gly49, Lys80, and Thr81 from H4. These interactions contribute to the positioning and stabilization of the 601–145 bp dsDNA around the histone octamer, supporting the preservation of the canonical nucleosome architecture despite sequence divergence among the histone variants.

### 3.3. Structural Analysis of Stacked AtNCPs

**Figure 3:**
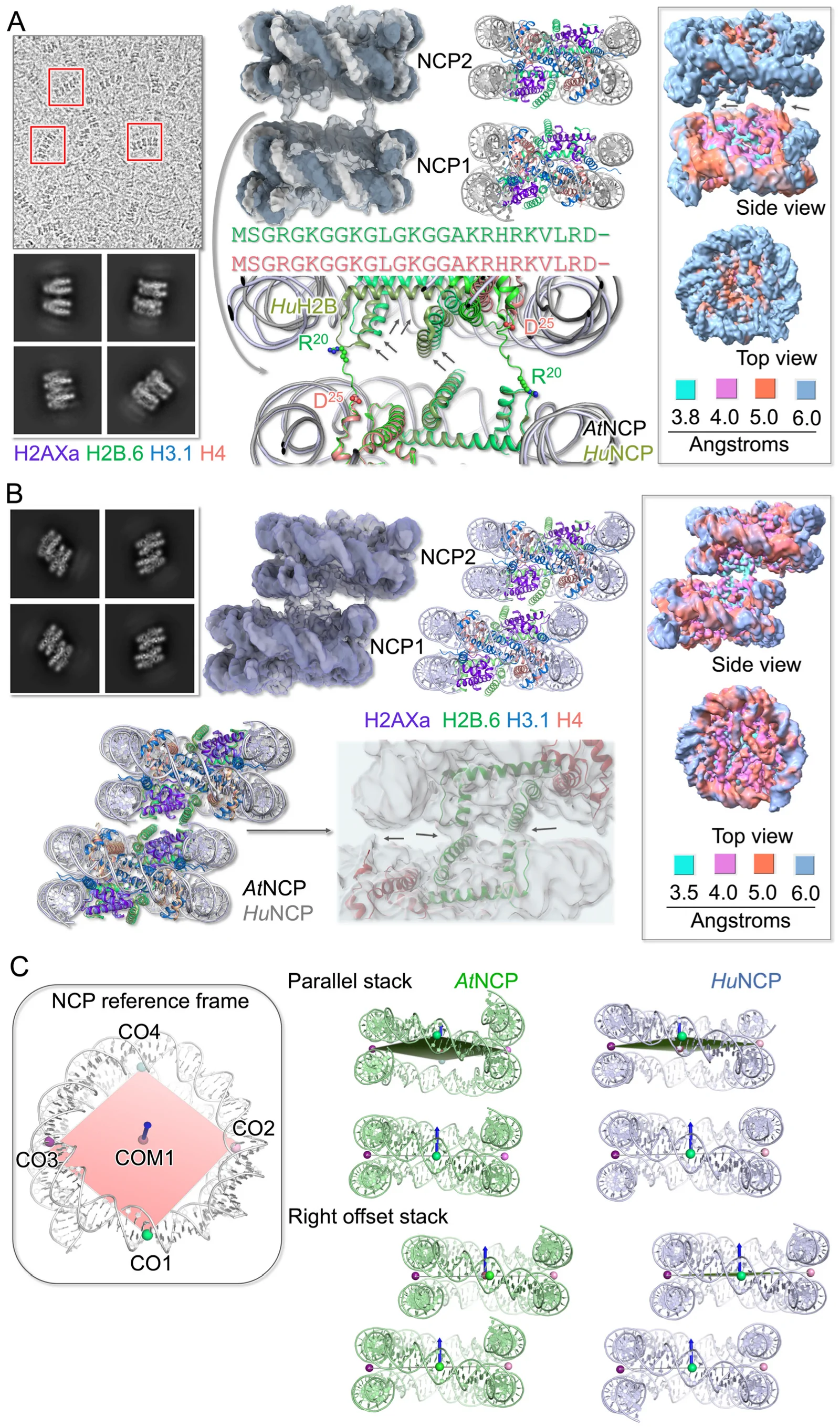
Analysis of nucleosome stacking in *At*NCP parallel and right-offset classes. (**A**) The cryo-EM density map and the model for the *At*NCP parallel stack. Zoomed-in cryo-EM grid images with stacked particles (boxed) and representative 2D class averages are shown. The EM density map and the corresponding models for the parallel stack are shown in the upper panel, with NCP1 and NCP2 indicated. H2AXa, H2B.6, H3.1, and H4 from Arabidopsis are shown in purple, green, blue, and salmon respectively. The local resolution map (inset, right) indicates that the EM density connecting NCP1 and NCP2 is resolved at approximately 6 Å. The limited resolution of the connecting density in the *At*NCP parallel stack precluded reliable modeling of the H4 N-terminal tail. The lower panel provides a zoomed-in view of the NCP-NCP interface, comparing the *At*NCP and previously determined *Hu*NCP parallel stack (PDB: 9MLL) models. H4 models and the corresponding N-terminal 25 residues in *Arabidopsis* (salmon) and human (green) are shown. Arrows indicate the concerted movement of the H2B structural elements of NCP2 from *Arabidopsis* (green) and human (smudge). These H2B structural elements of NCP2 are positioned to interact with H4 of NCP1 in the *Hu*NCP. (**B**) The cryo-EM density map and the model for the *At*NCP right-offset class. Representative 2D class averages of the right-offset stack are shown on the left. The EM density map and corresponding models of NCP1 and NCP2 are shown in the upper panel. The local resolution density maps shown in the right inset indicate that the NCP-NCP interface density is resolved at approximately 3.5–4 Å. The lower panel shows the *At*NCP right-offset stack superimposed with *Hu*NCP (PDB: 9MMO). The EM density and the model of the interface are shown with arrows. (**C**) Reference-frame construction for comparative geometric analysis of NCP-NCP stacking for the *At*NCP and *Hu*NCP. The inset shows the coordinates (CO_1_, CO_2_, CO_3_, and CO_4_) used for constructing the NCP plane (in salmon). The center of mass (COM) of the NCP was calculated for the coordinates of the double-stranded DNA. CO_1_ was defined as the centroid of the central base pairs of the 601–145 bp DNA, while CO_2_ and CO_3_ were defined from the centroid of the 19th base pair upstream and downstream of CO_1_. The centroid CO_4_ was defined from DNA bp diagonally opposite to CO_1_. The reference plane of the NCP was constructed using CO_1_, CO_2_, CO_3_, CO_4_ and COM, with the X- and Y-axes lying within the plane and the Z-axis oriented perpendicular to it. This NCP-centered reference frame was used to calculate the translational and rotational parameters describing NCP-NCP stacking. *At*NCP and *Hu*NCP stacks are represented in green and blue–white respectively.

Although the reconstituted sample was designed to generate mononucleosomes, the *At*NCP particles formed higher-order assemblies on the cryo-EM grids, with densities that could be resolved into distinct stacked nucleosome particles. Two major stacking arrangements were identified, corresponding to parallel and right-offset configurations (Figure 3A,B). The nominal resolutions for the two stacked nucleosomes, estimated using the gold-standard FSC 0.143 criterion, were 3.75 Å for the parallel stack and 3.45 Å for the right-offset stack (Supplementary Figure S1B,C). Local resolution analysis of the two stack structures indicated resolutions of approximately 3.5–5.0 Å for the histone cores and 5–6 Å for the superhelical DNA. In the parallel stack, the density connecting NCP1 and NCP2 was resolved at approximately 6 Å, precluding reliable modeling of the H4 N-terminal tail (Figure 3A).

In our previous cryo-EM studies on the human NCP parallel stack (*Hu*NCP; PDB: 9MLL), the H4 N-terminal tail was resolved and, together with structural elements of H2B, contributed to the NCP-NCP interface [13]. In the *At*NCP parallel stack, the corresponding inter-NCP density was not of sufficient resolution to allow reliable modeling of the H4 N-terminal tail (Figure 3A, right panel). Nevertheless, comparison with the *Hu*NCP parallel stack revealed a conserved overall organization of the inter-NCP interface, in which H2B structural elements from one NCP are positioned toward H4 of the opposing NCP. Comparative analysis of the *Hu*NCP and *At*NCP parallel stacks further revealed a displacement of the H2B structural elements in *At*NCP2 relative to the corresponding elements in *Hu*NCP2 (Figure 3A, bottom panel), indicating a plausible rearrangement of the H2B-mediated inter-NCP contacts in the plant NCP.

A similar comparative analysis of the *At*NCP and previously determined *Hu*NCP right-offset stacks (PDB: 9MMO) revealed a high degree of structural similarity, with an overall RMSD of 0.65 Å. The extensive NCP-NCP interface was well resolved at approximately 3.5–4.0 Å and exhibited a similar overall architecture in the two structures (Figure 3B). In both assemblies, the NCP-NCP interface was mediated primarily by structural elements of H2B, indicating that the organization of this higher-order interface is strongly conserved despite sequence divergence among the corresponding histone variants.

To quantitatively characterize the two *At*NCP stacking arrangements, we established an NCP-centered reference frame based on DNA coordinates of the canonical *At*NCP and calculated the relative orientation of the stacked NCPs. This reference-frame-based analysis enables the determination of translational and rotational parameters, including shift, slide, rise, interplanar angle, dihedral angle, inter-NCP distance, tilt, roll, and twist (Table 2) [13]. We next compared these stacking arrangements with those of the corresponding parallel and right-offset NCP assemblies previously identified in our study of human H2AX-containing nucleosomes (PDBs: 9MLL and 9MMO) [13] using the same reference-frame-based geometric analysis. The *At*NCP right-offset arrangement showed geometric parameters closely related to those of the corresponding *Hu*NCP structure, particularly for slide, rise and inter-NCP distance (Table 2, Figure 3C). In contrast, the *Hu*NCP parallel stack exhibited higher negative slide and roll values relative to the *At*NCP arrangement, consistent with the displacement of the H2B structural elements in NCP2 observed in the structural comparison (Table 2, Figure 3A, bottom panel).

Together, these analyses show that the *At*NCP forms parallel and right-offset stacking arrangements similar to those observed for human NCPs, with subtle differences in H2B positioning at the parallel inter-NCP interface.

## 4. Discussion

In this study, we provide the first structural characterization, to our knowledge, of an *Arabidopsis thaliana* nucleosome containing the replication-associated histones H2B.6 and H3.1 together with the DNA damage-responsive histone variant H2AXa. This distinct histone composition provides a structural framework for understanding chromatin organization in the context of DNA replication and repair, where ongoing DNA replication increases susceptibility to DNA damage and requires coordinated DNA replication and repair. Our model brings together these histone variants in a defined chromatin context; whether this specific histone combination occurs in vivo remains to be established and warrants further investigation.

Despite reconstituting mononucleosomes with defined 601–145 bp DNA, we observed pronounced nucleosome–nucleosome stacking in our cryo-EM dataset. Previous structural studies have commonly used tandem nucleosomal DNA templates, often incorporating the Widom 601 sequence, to generate arrays of nucleosomes and facilitate their ordered stacking [32,33,34,35,36]. In addition, chemical crosslinking has been employed in some studies to capture or stabilize inter-nucleosomal interactions [37], while protein factors can promote or stabilize nucleosome stacking [38]. In contrast, our preparation contained only recombinantly prepared mononucleosomes without linker DNA and devoid of additional protein factors or crosslinkers that could promote or disrupt nucleosome–nucleosome association. The observation of stacked particles under physiological conditions without additives therefore suggests that the reconstituted NCPs possess an intrinsic propensity to engage in defined inter-nucleosomal interactions in the absence of an extended nucleosomal DNA array or additional stabilizing factors. This is consistent with previous cryo-EM and biochemical studies showing that isolated NCPs can form trans interactions and that histone-mediated contacts contribute to nucleosome association [37,39]. In contrast to previous reports, our work presents the first subset structures of nucleosome array-like assemblies formed directly on cryo-EM grids from mononucleosomes, containing plant histone variants. Similar assemblies were also observed in our previous study of human nucleosomes [13].

Importantly, the stacked particles in our preparation adopted two distinct arrangements, corresponding to parallel and right-offset configurations. Similar stacking configurations were observed in our previous cryo-EM analysis of human H2AX- and γH2AX-containing nucleosomes [13]. In a broader analysis of 666 nucleosome structures deposited in the Protein Data Bank (PDB) as of September 2024, we found that these stacking modes recur across structurally characterized nucleosomes, which were primarily reconstituted with *Xenopus* histones. Together with the observation of these two stacking modes in both human and *Arabidopsis* nucleosomes, these findings indicate that the parallel and right-offset packing arrangements are not restricted to a single histone sequence or a single type of nucleosome species. Importantly, the occurrence of multiple defined stacking arrangements in our mononucleosome preparation further highlights the structural plasticity of the nucleosome and its capacity to engage in distinct modes of inter-nucleosomal association in the absence of extended linker DNA. These observations demonstrate the intrinsic propensity of reconstituted nucleosomes to form defined inter-nucleosomal contacts under the conditions used in this work, highlighting potential structural principles that may underlie nucleosome organization in plant chromatin. Further in vivo studies will be required to investigate this possibility in the plant context.

Despite sequence divergence, particularly in H2B.6 and H2AXa, the *At*NCP retains the canonical histone-fold architecture and extensive histone–DNA contacts characteristic of the nucleosome. This structural conservation is consistent with the central role of the histone-fold domains in maintaining the architecture of the nucleosome, while sequence-variable regions, particularly terminal tails and exposed surfaces, provide opportunities for variant-specific interactions. The ability of divergent histone variants to preserve the canonical nucleosome architecture has been observed in structural studies of specialized nucleosomes containing histone variants, including H2AZ, H2B.W2, Centromere Protein A (H3 variant), and H3.8 [40,41,42,43,44].

The higher-order structures further demonstrate that sequence variation in plant histones does not preclude formation of defined NCP-NCP interfaces. The *At*NCP right-offset stack closely resembles the corresponding human H2AX-containing NCP structure, with an overall similar slide, rise, and inter-NCP distance. The extensive interface in both structures is facilitated by H2B structural elements. Such H2B-mediated contacts have been observed in several nucleosome stacking arrangements and can occur together with H4 N-terminal tail and nucleosomal DNA [13,39,45]. The parallel *At*NCP assembly provides an additional point of comparison with its *Hu*NCP counterpart. While the H4 tail is not sufficiently resolved in the *At*NCP for reliable modeling, unlike the *Hu*NCP, the overall organization of the interface shows similarities to the H2B-H4-mediated architecture observed in the human structure [13]. However, the present density does not permit assignment of specific H2B-H4 contacts in the *At*NCP parallel stack structure.

The comparison of the two stacking arrangements further illustrates that nucleosome packing is not defined by a single geometry. The close correspondence between the plant and human right-offset assemblies contrasts with the more evident structural differences between their parallel arrangements. Rather than indicating a fundamentally different mode of nucleosome association, these differences reflect the ability of neighboring NCPs to adopt an alternative relative orientation while maintaining defined histone-mediated interfaces. Our reference-frame-based geometric analysis provides a quantitative description of these orientations for the *At*NCP and places the plant structures within the broader structural landscape of nucleosome packing.

The structures presented in this study represent the unphosphorylated state of H2Xa, with the C-terminal region containing the conserved SQ motif being unresolved due to intrinsic disorder. In our previous study of phosphorylated H2X-containing human nucleosomes, we observed that nucleosome stacking occurred irrespective of phosphorylation status, whereas binding of a phospho-H2AX reader protein to the phosphorylated H2AX tail disrupted the stacked arrangement [13]. These findings raise the possibility that phosphorylation-dependent reader interactions may similarly modulate nucleosome organization in plants; however, further in vitro and in vivo studies are required to determine how plant-specific reader proteins interact with nucleosomes and influence chromatin organization during DNA damage signaling.

Together, our structures demonstrate that the distinct histone composition of the *At*NCP is compatible with preservation of the canonical nucleosome architecture and formation of defined higher-order assemblies.

## Figures and Tables

**Table 1:** Cryo-EM data collection statistics.

Data Collection and Processing	Arabidopsis H2AXa Containing Nucleosome, Canonical (EMDB-78689) (PDB: 38BL)	Arabidopsis H2AXa Containing Nucleosomes, Parallel Stack (EMDB-78694) (PDB 38BO)	Arabidopsis H2AXa Containing Nucleosome, Right Offset Stack (EMDB-78669) (PDB 37ZO)
Magnification	105,000×	105,000×	105,000×
Voltage (keV)	300	300	300
Electron exposure (e–/Å^2^)	50	50	50
Defocus range (μm)	0.8–3.0	0.8–3.0	0.8–3.0
Angstrom per pixel (Å)	0.86	0.86	0.86
Symmetry imposed	C1	C1	C1
Final particle images (no.)	646,105	82,344	592,900
Map resolution (Å) FSC threshold	2.73 Å	3.75 Å	3.45 Å
Refinement			
Initial model used (PDB code)	9MMK	9MMK	9MMK
Map sharpening *B* factor (Å^2^)			
Model composition Non-hydrogen atoms Protein residues Nucleotide Ligands	11,816 747 290 -	23,632 1494 580 -	223,632 1494 580 -
R.m.s. deviations Bond lengths (Å) Bond angles (°)	0.006 0.916	0.006 0.917	0.006 0.918
Validation MolProbity score Clashscore Poor rotamers (%)	0.98 1.56 1.29	1.07 2.14 1.29	0.98 1.42 1.37
Ramachandran plot Favored (%) Allowed (%) Disallowed (%)	98.77 0.96 0.27	98.77 0.96 0.27	98.77 0.96 0.27

**Table 2:** Comparative geometric analysis of corresponding NCP-NCP stacking arrangements in human and Arabidopsis NCPs.

Structure IDs	Translational Parameters (Å)						Rotational Parameters (°)		
	Shift	Slide	Rise	Interplanar (°)	Dihedral (°)	Distance (Å)	Tilt	Roll	Twist
*At*NCP right offset (this study)	3.85	29.93	58.60	8.40	−2.96	65.92	0.42	2.80	7.96
HuNCP right offset (PDB: 9MMO)	6.29	30.88	58.93	10.39	−1.63	66.83	2.94	3.79	9.80
*At*NCP parallel (this study)	1.15	1.58	69.64	1.33	2.80	69.67	2.83	0.12	1.21
*Hu*NCP parallel (PDB: 9MLL)	0.25	−5.92	63.43	2.32	−2.65	63.70	1.71	3.27	0.09

## Data Availability

The atomic coordinates and corresponding cryo-EM density maps are available in the Protein Data Bank (PDB) and Electron Microscopy Data Bank (EMDB), respectively, with the following accession codes: PDB ID 38BL, EMD-78689, Arabidopsis H2AXa containing nucleosome, Canonical; PDB ID 38BO, EMD-78694, Arabidopsis H2AXa containing nucleosomes, Parallel stack; PDB ID 37ZO, EMD-EMD-78669, Arabidopsis H2AXa containing nucleosome, Right offset stack.

## Supplementary Materials

The following supporting information can be downloaded at https://www.mdpi.com/article/10.3390/cells15191795/s1. Figure S1: Validation of *At*NCP reconstructions using cryoSPARC.

## Abbreviations

The following abbreviations are used in this manuscript:

At	Arabidopsis thaliana
Hu	Human
NCP	Nucleosome core particle
EM	Electron microscopy
