# Supplementary material for: Molecular Architecture of the H2AX-Containing Nucleosome from Arabidopsis
Source: Cells. 2026 Oct 1;15(19):1795. doi: 10.3390/cells15191795 (PMC13664114; doi:10.3390/cells15191795)
Supplement: Supplementary file 1 [file cells-15-01795-s001.zip › FigureS1.pdf]

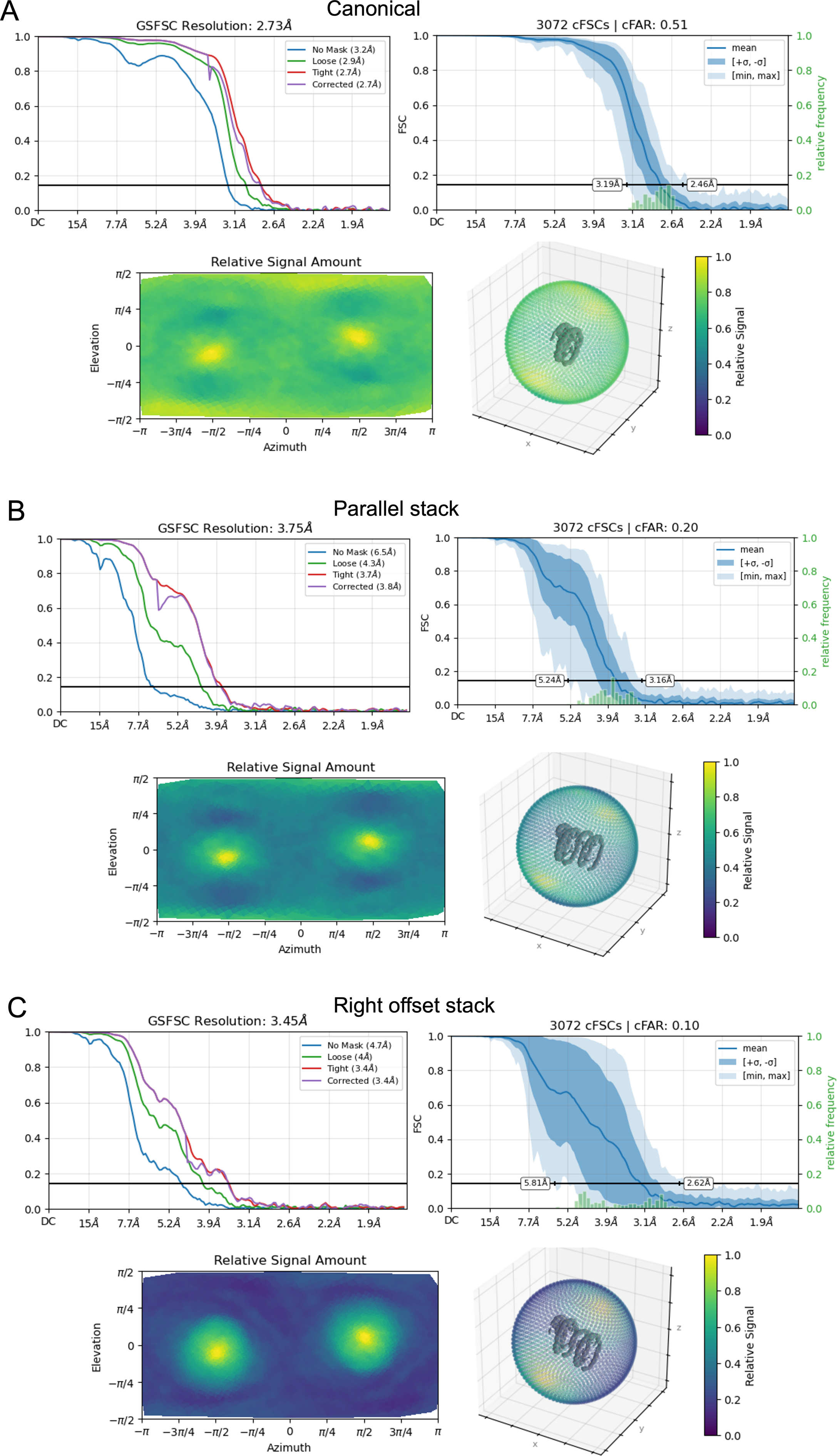
A
Canonical
GSFSC Resolution: 2.73Å
No Mask (3.2Å)
Loose (2.9Å)
Tight (2.7Å)
Corrected (2.7Å)
3072 cFSCs | cFAR: 0.51
mean
[+σ, -σ]
[min, max]
FSC
relative frequency
3.19Å
2.46Å
DC
15Å
7.7Å
5.2Å
3.9Å
3.1Å
2.6Å
2.2Å
1.9Å
Relative Signal Amount
Elevation
Azimuth
Relative Signal
B
Parallel stack
GSFSC Resolution: 3.75Å
No Mask (6.5Å)
Loose (4.3Å)
Tight (3.7Å)
Corrected (3.8Å)
3072 cFSCs | cFAR: 0.20
5.24Å
3.16Å
C
Right offset stack
GSFSC Resolution: 3.45Å
No Mask (4.7Å)
Loose (4Å)
Tight (3.4Å)
Corrected (3.4Å)
3072 cFSCs | cFAR: 0.10
5.81Å
2.62Å
